# Tall cell carcinoma with reversed polarity of breast cancer: a rare case report and review of the literature

**DOI:** 10.3389/fonc.2025.1635976

**Published:** 2025-11-03

**Authors:** Wenqi Duan, Longmei Zhang, Jinqi Gao, Zhe Wang, Mengjie Ren, Huajian Wu, Ya Deng, Chunlong Guo, Jianing Jiang

**Affiliations:** ^1^ Department of Radiation Therapy, Affiliated Zhongshan Hospital of Dalian University, Dalian, China; ^2^ Department of Breast Surgery, Dalian Women and Children's Medical Group, Dalian, China; ^3^ Department of Intervention, The Second Hospital Affiliated to Dalian Medical University, Dalian, China

**Keywords:** tall cell carcinoma with reversed polarity, TCCRP, TAM, *PI3K* and *IDH2*, precise treatment

## Abstract

Tall cell carcinoma with reversed polarity (TCCRP) is a primary invasive breast cancer that is clinicopathologically characterized by reversed polarity of the nucleus of the tumor cells near the cell edge. This pathologic feature is so rare that only 82 cases of TCCRP have been reported worldwide since it was first reported in 2003. Herein, we present the 83rd case of TCCRP. Going one step further than the previous reports, our case includes genetic testing. We sought to explore whether there are new mutation sites, with a view to identifying more therapeutic options for patients in future, to find PIK3CA p.H1047R mutation and IDH2 R172T mutation.We have found the macrophase makers CD68/CD163 double positive in this patient, which may indicate a poor prognosis in the early time. Therefore, the patient was treated with postoperative radiotherapy. The patient continues to be followed up for prognostic evaluation, and no recurrence or metastasis occurred until now. The case has represented a full view to gaining an improved understanding of TCCRP.

## Introduction

Tall cell carcinoma with reversed polarity (TCCRP) was first reported in 2003 and included as a histological subtype in the WHO breast cancer classification in 2019 (1). Morphologically, TCCRP resembles papillary carcinoma of the thyroid gland, with eosinophilic or hyaline cytoplasm of tumor cells, low-density ground-glass chromatin, as well as nuclear grooves and intranuclear pseudo-inclusions in some tumor cells. However, in immunohistochemistry studies, TCCRP exhibits no concordance with thyroid-specific markers Thyroid Transcription Factor-1(TTF-1), Thyroglobulin(TG), which proves that TCCRP lesions do not occur as a result of breast metastasis of papillary thyroid carcinoma. In addition, an interesting finding was “reversed polarity,” which refers to the arrangement of the tumor cell nuclei at the cell margins (2). In addition to the specific morphological features of TCCRP, the RET gene rearrangements and BRAF gene mutations typical of papillary thyroid carcinoma are not found in TCCRP (3, 4). Instead, *IDH2* R172S or R172T locus mutations are detected frequently (4), and, in a few cases, inactivating *TET2* mutations are detected, usually accompanied by gene mutations in the PI3K signaling pathway (2–5).

Because of the small number of cases worldwide, the mild clinical course, and the absence of specific signs and symptoms, the diagnosis of TCCRP is established on the basis of the histopathological feature of polarity reversal and is supplemented by genetic testing (6, 7), which is not mandatory. However, when the differential diagnosis between TCCRP and other types of papillary lesions of breast origin, such as solid papillary carcinoma, is not difficult or when the patient presents with a concurrent papillary thyroid carcinoma of indistinguishable origin, it would be necessary to depend on the specific molecular biology of hotspot mutations in the *PI3K* and *IDH2* genes for diagnosis.

Due to the rarity of the condition and good postoperative prognosis of most patients, only some patients are treated with adjuvant chemotherapy (7), with very few instances of lymph node metastases, distant metastases, and recurrences. Therefore, TRRCP is currently considered to be a unique (1, 2, 8), inert subtype of breast cancer, with low metastatic potential (3, 9). However, in the current case, we detected *PIK3CA p.H1047R* mutation as well as *IDH2 R172T* mutation (5), which further confirms the significance of the *PIK3CA* mutation site corresponding to the *PIK3* and *mTOR* pathways for immunotherapy. Although the condition is rare and the prognosis is generally good in most cases, there have been some cases of distant and brain metastasis (10, 11). With this report, we sought to provide evidence for the therapeutic benefit of radiotherapy and targeted drugs in this condition.

## Case report

We report the 83^rd^ case of TCCRP, Han Chinese, in a 68-year-old female patient with triple-negative breast cancer, with last menstrual period at the age 45 years, and no family history of cancer. Moreover, she denied any history of smoking or alcohol consumption, any history of infectious diseases, any history of hormone replacement therapy or prolonged radiation exposure. The patient’s occupation was retired, with no specific occupational hazards identified. Physical examination upon admission revealed bilaterally symmetrical breasts without obvious masses or skin changes. No peau d’orange appearance, skin dimpling, nipple retraction, or deviation was observed. There was no nipple discharge or bleeding. Palpation identified no nodules, tenderness, or fixation to the skin or underlying tissues, with normal mobility. No obvious abnormalities were detected in either breast. The patient had undergone surgical removal of a fibroadenoma in the left breast in 2014. In 2022, a right breast mass was detected, and vacuum-assisted biopsy of the right breast mass was performed. The intraoperative frozen section showed a intraductal papilloma with atypical ductal hyperplasia in a portion of the right breast mass. Paraffin-based pathology following the biopsy showed a tall cell breast carcinoma, with reverse polarity of the right breast. Immunohistochemistry of the frozen section revealed that the lesion was ER (-), PR (-), Her2(-), with Ki-67 (+15%).

**Figure 1:**
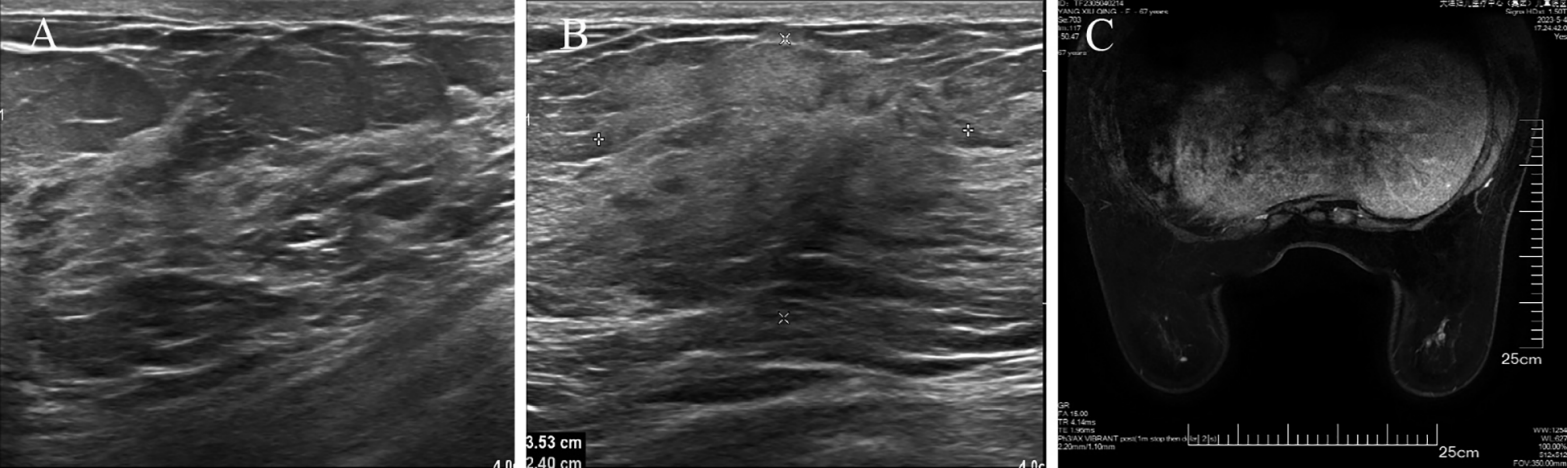
Preoperative imaging results of TCCRP. **(A, B)** Ultrasound imaging before the patient underwent a secondary procedure. **(C)** MR enhancement scan before the patient underwent a secondary procedure. Axial view of a T1-weighted contrast-enhanced fat-suppressed MR image obtained before the patient underwent a secondary procedure.

**Figure 2:**
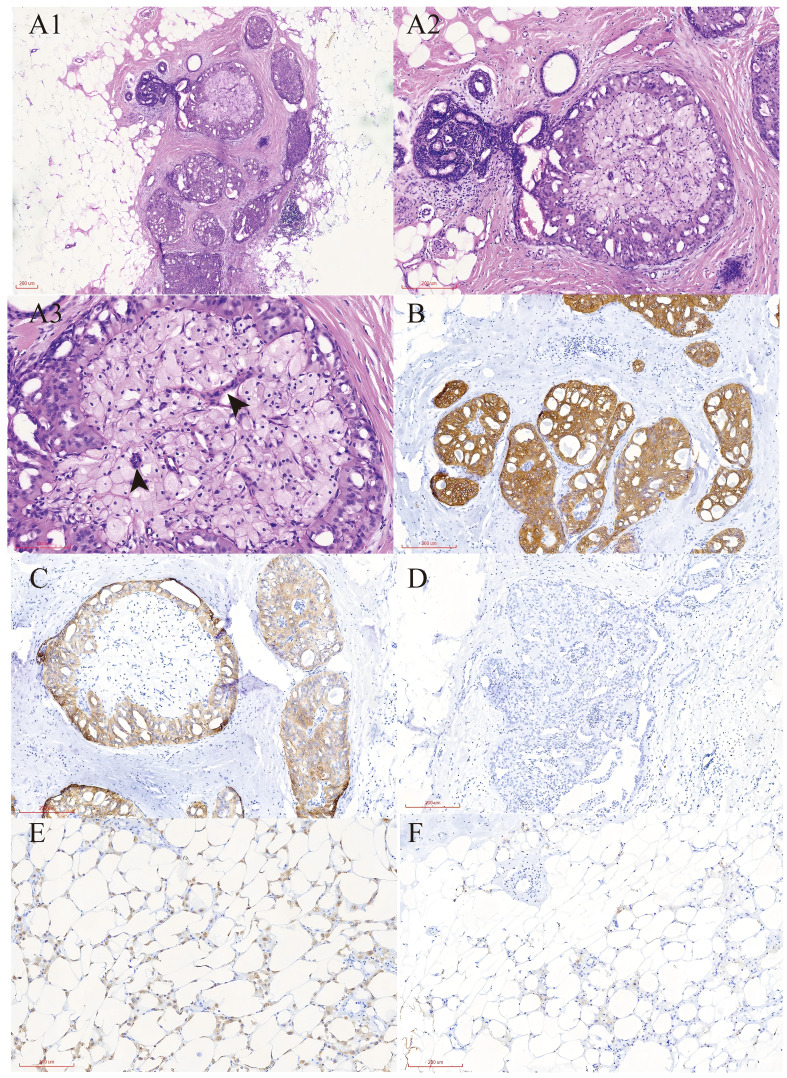
Pathological report of TCCRP. **(A)** Representative H&E stained sections show the histological features of tall cell carcinoma with reversed polarity. Magnification;A1×100,A2×200,A3×400. **(B)** Immunohistochemistry results show the cells were cytoplasmic positive for CK7.Magnification; ×100. **(C)** Immunohistochemistry results show the cells were cytoplasmic positive for CK5/6.Magnification; ×100. **(D)** Immunohistochemistry results show the cells were cytoplasmic positive for Ki67.Magnification; ×100. **(E)** Immunohistochemistry results show the cells were cytoplasmic positive for CD68.Magnification; ×100. **(F)** Immunohistochemistry results show the cells were cytoplasmic positive for CD163. Magnification; ×100.

**Figure 3:**
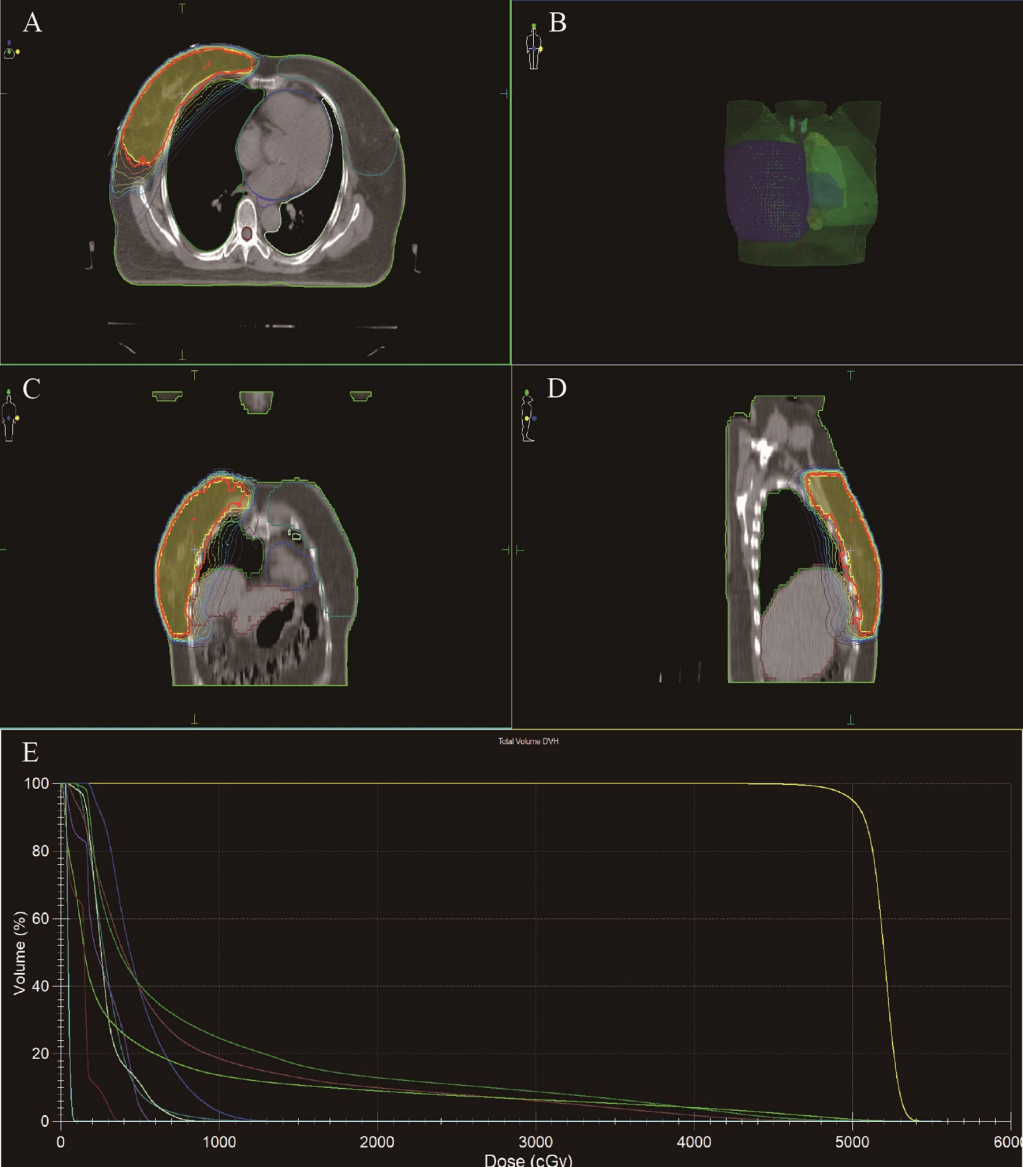
Radiotherapy target area outlining in TCCRP. **(A-D)** Radiation Treatment Target Delineation Image shows the target areas of tumor and normal tissues. **(E)** Total Volume DVH (DVH, Dose Volume Histogram) shows the distribution of radiation doses received by target areas of tumor and normal tissues. The DVH is a graphical representation of the radiation dose distribution delivered to the target volumes and critical organs at risk (OARs). The X-axis represents the radiation dose (in Gy). The Y-axis represents the volume of the structure (as a percentage) that receives at least the corresponding dose on the X-axis.

**Figure 4:**
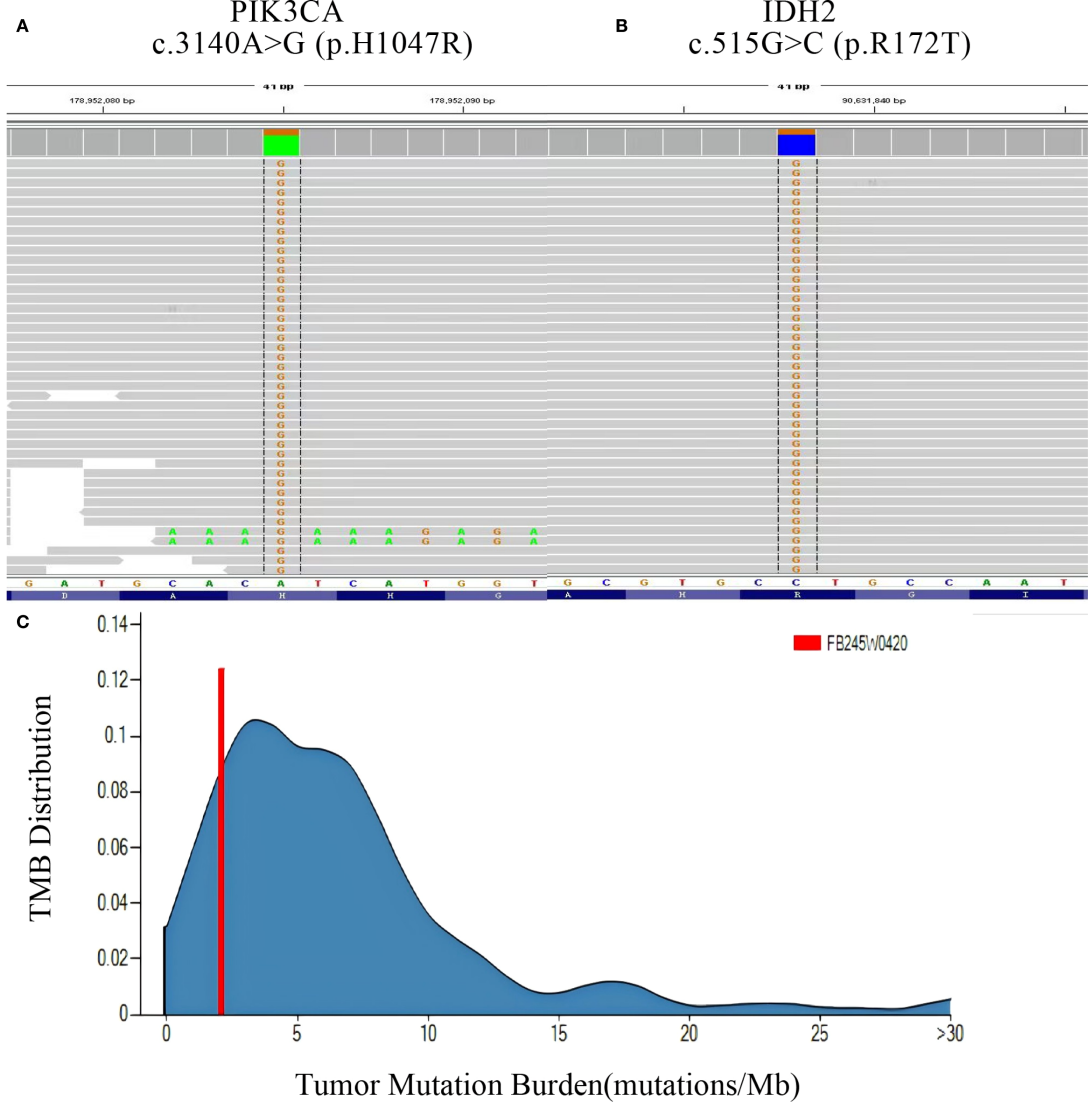
Panoramic genetic testing results of TCCRP. **(A, B)** NGS reads showing the mutation sites and abundance of PI3KCA and IDH2 genes. **(C)** TMB(Tumor Mutation Burden) reads showing the distribution range of TMB, respectively.

The patient underwent a secondary procedure to ensure negative margins. Preoperative ultrasound of the right breast showed slightly enhanced localized echogenicity at 7–9 o’clock, with a size of 3.5×3.2×2.4 cm, unclear borders, irregular morphology, no obvious blood flow signal on color Doppler flow imaging, and no obvious enlargement of lymph nodes in both axillae (**Figures 1A, B**). Contrast-enhanced magnetic resonance imaging (MRI) scan of the breast showed multiple scattered small nodular foci of significant enhancement in the right breast, with most of the lesions having a diameter of 2–3 mm and clear and smooth borders. One of the lesions was larger (diameter about 6mm) and located in the outer and lower quadrant, having a smooth border, with time-signal intensity curve (TIC) of the plateau pattern and significant enhancement of the ductal region seen beside it. The upper edge of the skin of the right breast was not smooth, and the reconstruction image showed a rounded area of slightly high signal intensity (**Figure 1C**). The right breast mass was then excised, and the upper, lower, inner, and outer margins of the excised tissue were collected and sent for examination. During the operation, and a bulky piece of breast tissue was seen, with size 7.5 × 6 × 1.7cm, and a grayish-yellow area was seen at the marking place, 0.8 × 0.6 × 0.5cm, with medium texture. The remaining breast tissue appeared grayish white, interspersed with areas of grayish yellow and was soft in texture. No cancerous tissue was seen at the margins of the excised breast tissue (upper, lower, inner, or outer). The tumor cells were arranged in a multinodular, solid, papillary arrangement, while the tumor cells themselves were hypercolumnar cytoplasmic, eosinophilic, and rich in mitochondria. Dispersed in the nodules were single round to oval mesonuclear grade cells. The oval nucleus showed invaginations of the nuclear membrane parallel to the long axis of the nucleus or a nuclear furrow with folded nuclear membrane, and the nuclear furrow invagination was encapsulated into the cytoplasm as intranuclear pseudo-inclusion bodies (**Figures 2A1-3**). The results of immunohistochemistry were as follows: ER (-), PR (-), HER-2 (-), Ki-67 (+ about 10%), AR (-), CK5/6 (+), CK7 (+), P63 (-), Calponin (-), SIA (-), SMMHC (-), GCDFP-15 (-), GATA-3 (-), TTF1 (-), CD68 (histiocyte+), CD163 (histiocyte+) (**Figures 2B–E**). On the basis of the investigative results, the patient was treated with local radiotherapy of the right breast after surgery, with planning target volume of 50Gy/25Fx (**Figures 3A–D**, **Table 1**). Dose Volume Histogram (DVH) shows, for any given point on a curve, the corresponding dose (X-value) and volume (Y-value) can be determined. For example, the CTV (Clinical Target Volume) curve shows that 100% of the volume received at least 36 Gy, and 95% of the volume (as per our planning goal) received the prescribed dose of 50 Gy. Steep curves (e.g., CTV) are ideal for target volumes, indicating a homogeneous dose with a sharp fall-off outside the target. Gradual curves (e.g., Lung-R) are typical for OARs(Off-Axis Ratio), showing that a gradient of doses is delivered across the organ. No acute adverse events of grade 2 or higher (according to CTCAE v5.0 criteria) during the radiotherapy was found. The patient was scheduled for periodic follow-up. This comprehensive surveillance strategy encompassed serial tumor marker evaluation (CEA, CA 125, CA 15-3), breast and abdominal ultrasonography, with the former including detailed inspection of the axillary, supraclavicular, and infraclavicular lymph nodes. Cross-sectional imaging via CT was utilized for the chest and head, supplemented by whole-body bone scintigraphy to screen for osseous metastases. Every 3 months for the first year, every 6 months for the second year and then annually thereafter. At the most recent follow-up visit in Aug, 2025 (approximately 24 months post-treatment), the patient remained asymptomatic with no clinical or radiological evidence of local recurrence or distant metastasis. The detailed timeline of key events related to the disease has been showed in **Table 2**. The resected cancer tissue was subjected to next-generation sequencing (NGS) using a GENESEEQPRIME^®^ pan-cancer panel covering approximately 1.5 Mb of the coding regions of over 400 genes (including exons, fusion-associated introns, variable shear regions, and specific microsatellite (MS) locus regions of more than 400 genes related to tumor targeting, diagnosis, prognosis, and tumor development). The TMB was calculated as 2.1 mutations/Mb, which is classified as TMB-Low based on the established cut-off of ≥ft mut/Mb for panels of this size. The results of NGS revealed that our patient had a missense mutation in exon 20 of the p.H1047R (PIK3CA), with a mutation abundance of 25.00%, and p.R172T exon 4 missense mutation in *IDH2*, with a mutation abundance of 21.91%. Additionally, 2.1 mutations/Mb, indicating a low tumor mutational burden (TMB), sorted in the top 84.59% were noted (**Figures 4A–C**). The mutational sites of enzymes related to polymorphisms in drug metabolism are as follows: *ERCC2* gene polymorphic mutation, *XRCC1* Q399R pure-sum mutation, *UGT1A1* polymorphic mutation, *TYMS*-6bp/-6bo polymorphic mutation, *CYP2B6* gene pure-sum polymorphic mutation, and *NQO1* gene polymorphic mutationality (**Table 3**). Mineralocorticoid receptor (MR)-related genes and MS-high (MSI-H) were not detected, and HLA-I typing was determined to be partially pure-sum (**Table 4**).

**Table 1:** The radiotherapy plans for tumor and normal tissues, covering specific doses.

Structure	Volume (cm3)	Min dose cGy	Max dose cGy	Mean dose cGy	Ref. vol. (%)	Ref. dose. cGy
CTV	1015.570	3615.0	5558.0	5183.6	95.00	5000.0
Lung-R	1383.025	0.0	5247.1	878.1	12.88	2000.0
	1383.025	0.0	5247.1	878.1	40.37	500.0
Lung-L	959.875	42.0	924.3	291.8	11.18	500.0
Heart	651.940	164.3	1404.3	490.6	0.00	3000.0
Esophagus	32.300	28.8	578.6	265.1	0.00	587.6
Liver	1299.425	35.6	5193.9	744.1	6.03	3000.0
Spinal Cord	40.630	12.1	377.0	132.2	0.00	377.0
Thyroid	7.380	27.1	91.6	48.3	0.00	5000.0
Contralateral Breast	1074.130	0.0	1248.2	302.3	0.01	1200.0

**Table 2:** Timeline of key clinical events for the reported TCCRP case.

Date/Time period	Event/Intervention	Key findings/outcome
~11 years prior	Surgical removal of left breast fibroadenoma	Benign; no evidence of malignancy
3 years prior	Detection of right breast mass; Vacuum-assisted biopsy	Intraductal papilloma with atypical ductal hyperplasia
Subsequent	Definitive surgical excision	Diagnosis of Tall Cell Carcinoma with Reversed Polarity (TCCRP); Clear margins
Post-operation	Immunohistochemistry analysis	ER(-), PR(-), HER2(-), Ki-67 (~10%); CD68+, CD163+
Regular follow-up	Next-Generation Sequencing (NGS) Adjuvant Radiotherapy Clinical and imaging surveillance	Detection of PIK3CA p.H1047R (25%) and IDH2 R172T (21.91%) mutations; TMB-Low (2.1 mut/Mb) Whole breast irradiation; 50 Gy/25 fractions; Well-tolerated No evidence of local recurrence or distant metastasis to date

**Table 3:** The mutation sites of enzyme polymorphisms related to drug metabolism.

Gene mutation	Chemotherapy drug for which sensitivity is predicted
Polymorphic mutations in the *ERCC2* gene	Platinum (cisplatin, carboplatin, oxaliplatin, etc.) may be less effective than wild type
*XRCC1* Q399R, pure-sum mutation	Efficacy of platinum-based drugs (cisplatin, carboplatin, oxaliplatin, etc.) may be better than that in case of the wild-type
Polymorphic mutations in the *UGT1A1* gene	Irinotecan, etoposide toxicity may increase
*TYMS*-6bp/-6bo polymorphic mutation	Pemetrexed, fluorouracil efficacy may be better than that in case of *TYMS* wild type
Pure and polymorphic *CYP2B6* gene *NQO1* gene polymorphism	Possible increase in cyclophosphamide toxicity Mitomycin may be less effective than that in case of wild type

**Table 4:** Comprehensive HLA-I typing assessment results predict efficacy against immune checkpoint inhibitors.

HLA-A	HLA-B	HLA-C	Comprehensive HLA-I typing assessment
A*02:01	B*13:01	C*01:02	partial pure
A*02:01	B*13:01	C*03:04	partial pure

## Discussion

Worldwide, breast cancer ranks first in incidence rate (23.8%) and mortality rate (15.4%), surpassing lung cancer. According to the latest report (cancer) released by The National Cancer Center of China (NCC), there were 357,200 new cases of breast cancer in 2022, accounting for 7.4% of the 4,824,700 new cancer cases. This shows that the incidence of breast cancer among the Chinese female population with cancer (2,290,800, cases) is the second highest, after lung cancer. Additionally, breast cancer is one of the top five causes of cancer-related mortality (12, 13).

In terms of molecular typing, the types of breast cancer recognized are as follows: luminal A (ER+/PR+,HER-2-), Luminal B (ER+/PR+,HER-2+), HER-2 overexpression (ER-/PR-/HER-2+), and Basal-like (ER-/PR-/HER-2-). Our patient had the basal-like (ER-/PR-/HER-2-) type. Although most of the molecular subtypes of TCCRP patients are triple-negative (5, 8), some patients present with tumors that are ER/PR weakly positive. Among the 83 patients reported thus far, TCCRP has shown no significant correlation with ethnicity and geography, and the age of onset of the disease ranges from 40 to 85 years.

The results of immunohistochemistry studies in this case were as follows: showed ER(-), PR(-), HER-2 (-), Ki-67(+ about 10%), AR(-), CK5/6(+), CK7(+), P63(-), Calponin(-), SIA(-), SMMHC(-), GCDFP-15(-), GATA-3(-), TTF1(-), CD68 (histiocyte+), and CD163 (histiocyte+). The diagnosis of TCCRP is determined not only by its distinctive morphology and molecular genotype, the variable expression of conventional breast markers. Among these markers, CD68 and CD163 are two important indicators that are related to the tumor microenvironment (TME). TME is a general term that refers to the environment in which tumor cells are located, and it is mainly composed of mesenchymal cells, neighboring cells, blood vessels, and immune cells, in addition to tumor cells. The TME can be divided into a non-immune microenvironment, which is dominated by fibroblasts and vascular endothelial cells, and an immune microenvironment, which is dominated by macrophages. The components of the TME, especially, the tumor-associated macrophages (TAMs), are the occurrence of breast cancer and tumorigenesis (14). This makes these important targets in the diagnosis and treatment of breast cancer. Leukocyte differentiation antigen molecule 68 (CD68) is considered to be one of the main markers involved in the activation and infiltration of TAMs, while CD68 is a characteristic marker of M1-type macrophages, which exert a tumor-killing effect caused by DNA damage, resulting from the production of oxygen free radicals, and inhibit tumor cell growth. CD163 is known to be a characteristic marker of M2-type macrophages, which are considered to be immune cells involved in tumor growth and metastasis, and its activities include the induction of angiogenesis, inhibition of T-cell activity, and release of chemokines such as inflammatory growth factors, for the promotion of tumor cell growth. Some studies have shown that the five-year progression-free survival (PFS) is higher in non-metastatic breast cancer patients with low expression of CD68-positive macrophages and that the prognosis of patients is worse when CD68 positivity is highly expressed (15). TAMs with double positivity for CD68 and CD163 are more likely to be found in lymph node metastatic tumors in the tumor microenvironment (5, 6). Macrophages with CD68/CD163 double positivity are associated with poor prognosis of early-stage breast cancer, high-level histological grading, and high Ki67 expression. Positivity of macrophages only for CD163 has been found to be associated with tumor stage and lymph node status (16), whereas macrophage positivity for only CD68 has been associated with the molecular subtype of breast cancer, independent of other clinicopathological parameters. In this patient with TCCRP, we found the presence of TAM M1-type and M2-type markers for the first time. During the short term of the postoperative follow-up, this patient has had good overall prognosis; however, continued follow-up for 10–15 years is still needed to assess the efficacy of the treatment as a whole. Most of the current evidence has shown that the presence of CD68/CD163 double-positive macrophages in the early stage represents a poor prognosis (13); therefore, after discussing the risks of the condition adequately with the patient, we opted to administer total breast radiation therapy to further minimize the risk of future recurrence (8, 12, 13, 17). Although the prognostic significance of TAMs in TCCRP remains undefined, the presence of a CD68+/CD163+ macrophage infiltrate, a signature associated with adverse features in broader breast cancer cohorts, was considered a potential high-risk factor in this individual case. Moreover, combined with the patient’s willingness for maximal local control after breast-conserving surgery, the patient was scheduled for adjuvant radiotherapy postoperatively. This highlights the necessity for larger studies to validate prognostic markers and define optimal adjuvant therapy in TCCRP.

In addition, the results of NGS showed that our patient had 2.1 mutations/Mb, which indicates a low TMB and for which immunotherapy was considered to be of little benefit. In addition, the patient had a tumor-specific mutation of the *PIK3CA* gene at exon 20, a missense mutation with mutation type c.3140A>G (p.H1047R), and mutation abundance of 25.00% as well as *IDH2* gene mutation P.R172T at exon 4, missense mutation with mutation type c.515G>C (p.R172T), and mutation abundance of 21.91%; this is a non-embryonic mutation that is not hereditary and is consistent with the existing findings. *PIK3CA p.H1047R* mutation is located in the structural domain of phosphatidylinositol 3/4 kinase and is a common activating mutation in the *PIK3CA* gene. *PIK3CA* has been implicated in various cancers, including gastric cancer, hepatocellular cancer, non-small-cell lung cancer, and breast cancer and it can activate the PI3K/AKT/mTOR signaling pathway by enhancing the activity of PI3K lipid kinase, thus promoting the invasive and metastasizing ability of cancer cells and contributing to tumor development. *PIK3CA*-activating mutations can increase the sensitivity of PI3K, mTOR inhibitors such as everolimus (18). In 2019, the FDA approved a regimen comprising the inhibitor apelalisib in combination with fulvestrant, targeting the PI3Kα isoforms, as well as the regimen of capivaserti in combination with fulvestrant, targeting three AKT isoforms to be used in patients with HR-positive, HER2-negative breast cancer harboring *PIK3CA* mutations. Fulvestrant has no estrogenic effect per se, but it can bind ER to competitively antagonize the estrogenic effect; when fulvestrant binds to ER, it can block ER function by inducing ER degradation. *IDH2* p.R172T mutation has been reported in osteoblastoma, chondrosarcoma, and hemolymphoma, and R172 is a hotspot mutation site of *IDH2* (12). Studies have shown that mutation in the R172 site can be used to treat patients with HR-positive HER2-negative breast cancer carrying PIK3CA mutation. The R172 locus mutation can influence *IDH2*-encoded protein isocitrate dehydrogenase activity by decreasing the levels of intracellular α-ketoglutarate, which may promote the conversion of isocitrate to 2-hydroxyglutarate (2-HG); The resultant overaccumulation of 2-HG causes excessive cellular proliferation, which, in turn, contributes to oncogenic changes and tumor growth and promotes tumor development (2). Some studies have shown that the *IDH2* inhibitor endesipine can be used to treat relapsed or refractory acute myelogenous leukemia carrying *IDH2* mutations. Considering these observations, we believe that it may be worthwhile to examine whether relapsed and metastatic TCCRP patients with PIK3CA-activating mutations and *IDH2* mutations would benefit from the drugs everolimus and endesipine. In future, we also intend to explore the effects of these drugs and their molecular mechanisms through more case studies and *in vitro* and *in vivo* investigations, so as to obtain more evidence regarding the targeted therapy of TCCRP (8, 10, 11, 19).

Furthermore, in this case, the results of NGS for the sensitivity of the tumor to common chemotherapeutic drugs revealed CYP2B6*6 polymorphism, i.e., the simultaneous occurrence of *p.Q172H* and *p.K262R* heterozygous mutations in both exons 4 and 5 of the *CYP2B6* gene; this, in turn, reduces the *in vivo* activity of the 2B6 enzyme and decreases its effects on drug metabolism, leading to increased toxic adverse effects of cyclophosphamide and isocyclophosphamide (9). The *p.K751Q* heterozygous mutation in the *ERCC2* gene is a single-nucleotide polymorphism (rs13181) missense mutation, which is associated with the risk of various tumors such as lung, bladder, breast, and skin cancers, and low risk indicators. Lung cancer patients carrying the *K751Q* mutation have been shown to be resistant to platinum drugs. The *p.P187S* pure mutation (i.e., the c.559C>T mutation) in the *NQO1* gene is a missense mutation caused by the single-nucleotide polymorphism rs1800566, with a risk allele of T. This mutation can lead to a loss of activity by affecting the enzyme’s stability, which has a protective function against benzene toxicity, is therefore disruptive. The mutation can increase the risk of breast cancer as well as lung cancer in non-smokers as well as the risk of hematotoxicity and leukemia in individuals with environmental exposure to benzene. In addition, this polymorphism may be associated with a poorer prognosis in breast cancer patients who have received doxorubicin and cyclophosphamide chemotherapy. The *TYMS* gene 6-bp pure deletion polymorphism (rs151264360) is a deletion mutation of a 6-bp (TTAAAG) nucleotide fragment in the 3-UTR region. This genotype reduces the level of expression of thymidylate synthase compared to the wild-type and enhances the expression of fluoride and the chemotherapeutic agent pemetrexed (20). The UGT1A1*28 polymorphism (rs34983651) is an A(TA)(6)TAA>A(TA)(7)TAA mutation triggered by TA insertion upstream of the gene. This heterozygous polymorphism significantly reduces the expression and activity of the *UGT1A1* enzyme, which increases the toxicity and adverse effects of irinotecan and etoposide (21). The *XRCC1* gene p.Q399R pure mutation is a missense mutation caused by single-nucleotide polymorphism rs25487, which is associated with the risk of non-small-cell lung cancer, breast cancer, colorectal cancer, gastric cancer, and other types of tumors. GG pure mutation is associated with the efficacy of platinum-based chemotherapeutic drugs and can enhance the response rate of cells to platinum-based drugs. Therefore, in this case of TCCRP, the patient had increased sensitivity to the drugs pemetrexed and fluorouracil, with increased toxicity to the drugs irinotecan, etoposide, and cyclophosphamide. These chemotherapeutic drug choices form the basis of the treatment for possible subsequent recurrence and metastasis. HLA-I typing was determined to be partially pure-sum, which hints that overall survival after immune checkpoint inhibitor treatment was lower for pure and partially pure HLA1 compared to total pure cases.

TCCRP is rare worldwide, has an inert pathological type, and progresses to advanced stages only in a few cases. However, it is important patients with TCCRP are monitored with long-term follow-up, to rule out the possibility of recurrence and metastasis. Currently, there is inadequate evidence to show that postoperative adjuvant radiotherapy is beneficial for patients with TCCRP. However, since our patient had positivity for both CD68 and CD163, which is considered to indicate poor prognosis and since the patient requested postoperative adjuvant radiotherapy, we irradiated the whole breast on the affected side, without subsequent dosing in the tumor bed area. The patient continues to be followed up, and until the time of preparation of this manuscript, she has not shown any signs of recurrence or metastasis. We believe that publication of further reports on similar cases and long-term follow-up of the patients will help us determine whether or not adjuvant radiotherapy can be beneficial.

In conclusion, our report not only adds to the evidence regarding the diagnosis of a rare pathotype, but also identifies, for the first time, the presence of CD68/CD163 in TCCRP. We have also discussed the chemosensitivity of different genetic loci involved in this condition as well as feasibility of using targeted drugs against the *PI3K* and the *IDH2* mutation loci in TCCRP. We have also employed postoperative adjuvant radiotherapy in this breast-conserving patient and followed up its effect on prognosis in this case, with a view to gain insight into the individualized and precise treatment of TCCRP. Our experience, in this case, may bring more prospective and cautionary evidence about TCCRP, and TAM, as an important target of immune checkpoint inhibitors, may trigger more new lines of investigation regarding immunotherapy for TCCRP.

## Data Availability

The data presented in the study are deposited in the NGDC repository, accession number HRA014091.

## Glossary

ER: Estrogen receptor
PR: Progesterone receptor
HER2: Human epidermal growth factor receptor 2
AR: Androgen receptor
TMB: Tumor mutational burden
TAM: Tumor-associated macrophages
CK5/6: Cytokeratin 5/6
CK7: Cytokeratin 7
P63: Tumor protein 63
SIA: Sialic acid
SMMHC: Smooth Muscle Myosin Heavy Chain
GCDFP-15: Gross Cystic Disease Fluid Protein-15
GATA-3: GATA Binding Protein 3
TG: Thyroglobulin
TTF1: Thyroid Transcription Factor-1
CD68: Cluster of Differentiation 68
CD163: Cluster of Differentiation 163
DVH: Dose Volume Histogram
CTV: Clinical Target Volume
OAR: Organs at risk
